# Avatrombopag for the treatment of thrombocytopenia in children's patients following allogeneic hematopoietic stem-cell transplantation: A pilot study

**DOI:** 10.3389/fped.2023.1099372

**Published:** 2023-02-15

**Authors:** Yongsheng Ruan, Wei Cao, Tingting Luo, Xuan Liu, Qiujun Liu, Yuhua Xiao, Cuiling Wu, Danfeng Xie, Yuqiong Ren, Xuedong Wu, Xiaoqin Feng

**Affiliations:** Department of Pediatrics, Nanfang Hospital, Southern Medical University, Guangzhou, China

**Keywords:** avatrombopag, thrombocytopenia, allogeneic hematopoietic stem cell transplantation, thrombopoietin receptor agonist, poor graft function, secondary failure of platelet recovery

## Abstract

Thrombocytopenia following allogeneic hematopoietic stem cell transplantation (allo-HSCT) is a common and life-threatening complication. Thus, new prevention and treatment strategies for post-HSCT thrombocytopenia are urgently required. In recent studies, thrombopoietin receptor agonists (TPO-RA) for treating post-HSCT thrombocytopenia indicated efficiency and safety. The improved effect of post-HSCT thrombocytopenia in adults was found in the administration of avatrombopag which was a new TPO-RA. However, there was no relevant study in the children's cohort. Herein, we retrospectively analyzed the effect of avatrombopag in post-HSCT thrombocytopenia in children. As a result, the overall response rate (ORR) and complete response rate (CRR) were 91% and 78%, respectively. Furthermore, both cumulative ORR and CRR were significantly lower in the poor graft function (PGF)/secondary failure of platelet recovery (SFPR) group compared to the engraftment-promotion group (86.7% vs. 100%, *p* = 0.002 and 65.0% vs. 100%, *p* < 0.001, respectively). Achieving OR required a median of 16 days in the PGF/SFPR group while 7 days in the engraftment-promotion group (*p* = 0.003). Grade III–IV acute graft vs. host disease and inadequate megakaryocytes were identified as risk factors of CRR only in univariate analysis (*p* = 0.03 and *p* = 0.01, respectively). No severe adverse events were documented. Conclusively, avatrombopag is an alternatively efficient and safe agent for treating post-HSCT thrombocytopenia in children.

## Introduction

Thrombocytopenia after allogeneic hematopoietic stem cell transplantation (allo-HSCT) is a common complication and may lead to increased transplant-related mortality (TRM) (1–3). Specifically, prolonged thrombocytopenia, a platelet count <100 × 10^9^/L on day +100 post-transplant, has been identified as an independent risk factor of elevated TRM (2, 4). The etiology of thrombocytopenia post-transplant consists of multiple factors mainly including poor graft function (PGF), graft vs. host disease (GVHD), infections, HLA-mismatched transplant, medications, immune-related consumption, bone marrow microenvironment, and microangiopathy (1, 5). Based on the time of thrombocytopenia occurrence, PGF, secondary failure of platelet recovery (SFPR), and prolonged isolated thrombocytopenia (PIT) are well defined recently (1, 6, 7). Therefore, new prevention and treatment strategies are urgently required.

Nowadays, the treatment strategies for post-transplant thrombocytopenia include (1) removal of potential causes mentioned previously, (2) support care such as transfusion of platelet, (3) various agents promote the recovery of platelets like thrombopoietin receptor agonists (TPO-RA), recombinant human thrombopoietin (rhTPO), and mesenchymal stem cells (MSC) (3, 6). Furthermore, eltrombopag and romiplostim as TPO-RA have been widely investigated in thrombocytopenia post-transplant and indicated well-tolerance and efficiency (1, 8, 9). However, to date, there have been only a few studies for avatrombopag in the post-HSCT thrombocytopenia setting. Avatrombopag, a relatively new TPO-RA, has been approved for the treatment of thrombocytopenia in adults with chronic liver disease by the U.S. FDA (10, 11). Additionally, avatrombopag is more feasible with or without food intake features than eltrombopag (11). Therefore, avatrombopag may potentially be appropriate for pediatric patients. Thus, we implemented a pilot retrospective study to determine the efficacy and safety of avatrombopag for thrombocytopenia in children's patients following allo-HSCT.

## Methods

### Patients

Between September 2021 and October 2022, 30 patients who underwent allo-HSCT in the Department of Pediatrics, Nanfang Hospital, Southern Medical University were enrolled. Inclusion criteria were defined for those patients who had PGF, SFPR, or PIT with complete engraftment of donor cells. Exclusion criteria were defined for patients with leukemia relapse or abnormality of hepatic function (more than 1.5 times the upper limit of normal serum glutamate pyruvate transaminase and bilirubin). A total of 32 thrombocytopenia events occurred and were treated with avatrombopag in these patients. The institutional review board at Nanfang Hospital hospital approved the retrospective study, and all consent forms approved by the institution were signed.

### Transplant protocols

The donor selection and the transplant protocol were reported in previous studies (12–14). In general, busulfan, cyclophosphamide, and fludarabine with or without thiotepa were administrated as a myeloablative conditioning regimen for both HLA-matched and HLA-mismatched patients. Posttransplantation cyclophosphamide (PTCY) with or without fludarabine at day +3 and day +4 was utilized as a haploidentical transplant regimen.

### Definitions

PGF was defined as persistent neutropenia (absolute neutrophil count <0.5 × 10^9^/L), thrombocytopenia (platelets <20 × 10^9^/L), and/or hemoglobin <70 g/L for at least 3 consecutive days by day 28 post-HSCT with transfusion requirement in the presence of complete donor chimerism without disease relapse (15, 16). SFPR was defined as platelets <20 × 10^9^/L for 7 consecutive days or requirement of transfusion following reaching platelets ≥50 × 10^9^/L without transfusion for 7 days post-HSCT (17). Meanwhile, the promotion of platelet engraftment was proposed to facilitate platelets ≥20 × 10^9^/L without transfusion for 7 days post-HSCT. The number of megakaryocytes in bone marrow (BM) smear less than 7 in 1.5 cm × 3.5 cm area was considered as inadequate megakaryocyte status (18, 19).

### Administration of avatrombopag

Since this was a retrospective pilot study, randomization, blinding, and power analysis were not applicable. Avatrombopag was administrated for the patients with PGF or SFPR. It was indicated for engraftment-promotion patients who had a history of immune cytopenia, immune autoantibodies detected positive post-HSCT (direct Coomb's test or platelet antibody test), or encountered hemorrhage complications post-HSCT. The initial dosage was 10 mg once a day for a weight of less than 30 kg patients while 20 mg for ≥30 kg patients. Subsequently, the elevated dosage by an interval of 10 mg every two weeks according to the response with a maximum dosage of up to 40 mg/day (Supplementary Table S1). Avatrobopag was tapered or ceased if the platelet count achieved ≥50 × 10^9^/L or 100 × 10^9^/L, respectively, without transfusion. The overall response (OR) was defined as the platelet ≥20 × 10^9^/L for at least 7 consecutive days without transfusion. Meanwhile, the complete response (CR) was defined as the platelet ≥50 × 10^9^/L for at least 7 consecutive days without transfusion (18). Adverse events and severe adverse events were evaluated according to the National Cancer Institute Common Terminology Criteria for Adverse Events (NCI-CTCAE) version 5.0.

### Statistical analyses

Two-tailed *t*-test or Kruskal–Wallis test were performed for continuous variables between groups. Categorical variables were compared using the *χ*^2^ test or Fisher's exact test. The univariate and multivariate logistic regression analyses were conducted to identify risk factors of treatment of avatrombopag. The factors in univariate analysis with a *p*-value < 0.05 were input into multivariate analysis. The cumulative incidence of OR and CR was calculated by a competing risk model. The overall survival probability (OS) was determined by the Kaplan–Meier analysis and compared with the log-rank test. It was considered statistically significant if the *p*-value <0.05. Data analyses were completely conducted with RStudio (version 2022.02.0 + 443).

## Results

### Patient characteristics and outcomes

**Figure 1:**
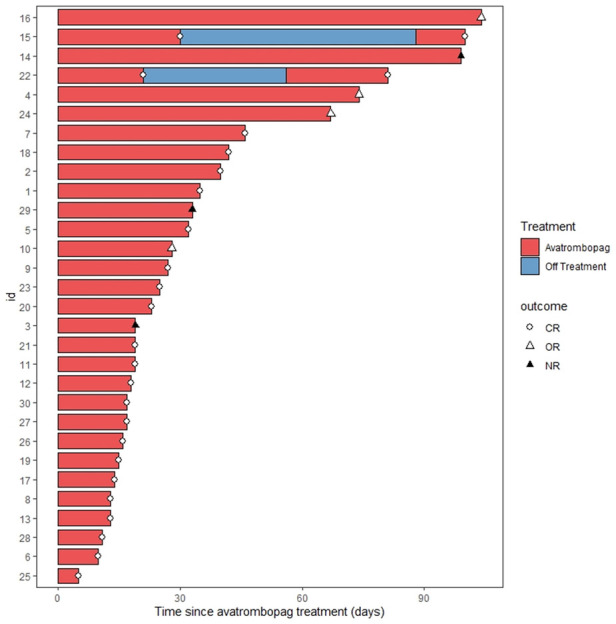
Swimmer plot for the response evaluation in all patients. CR, complete remission; OR, overall response; NR, no response.

**Figure 2:**
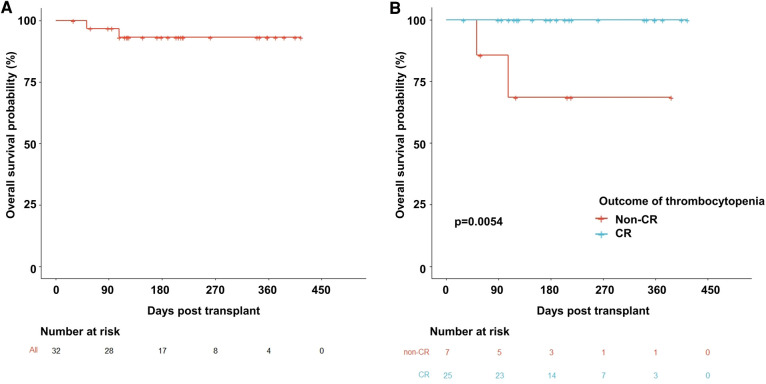
Overall survival probability curves. (**A**) The whole population used avatrombopag. (**B**) Comparison between the non-CR and CR groups. CR, complete remission.

A total of 30 patients with 32 thrombocytopenia events were included in this pilot study (Figure 1). The details of the transplant were summarized in Table 1. The median age of the patients was 6 years old. Thalassemia major (TM) accounted for 59% of the disease composition. All patients received myeloablative conditioning. In addition, all patients were treated with avatrombopag as indicated, including 15 (47%) patients with PGF, 5 (16%) patients with SFPR, and 12 (37%) patients with the aim of engraftment promotion. No one PIT patient was documented during the study period. In general, 56% of patients underwent HLA-mismatched transplants with 31% cord blood engraftment. PTCY was the main transplant regimen which accounted for 88%. The median days of neutrophil and platelet recovery were 21 days and 18 days, respectively. Eleven (34%) patients and three patients suffered from III–IV acute GVHD (aGHVD) and extensive chronic GVHD (cGVHD), respectively. Sixteen (50%) patients experienced CMV infection. Autoimmune hemolytic anemia (AIHA) and immune thrombocytopenia (ITP) occurred in 19 (59%) and 4 (12%) patients, respectively. Five (16%) patients had veno-occlusive disease (VOD). Two (6%) patients underwent transplant-associated thrombotic microangiopathy (TA-TMA). Hemorrhagic cystitis was the most common hemorrhage event accounting for 12 (38%) patients. Moreover, the overall survival probability (OS) was 93.2% ± 4.7% [95% CI (84.5%–100%)] (Figure 2A).

**Table 1:** Characteristics of patients.

Characteristic	Overall, *N* = 32	No response, *N* = 7	Complete response, *N* = 25	*p*-value
Age [year, mean (SD)]	6.44 (3.29)	7.43 (2.64)	6.16 (3.45)	0.3
Sex				0.076
Male	23 (72%)	3 (43%)	20 (80%)	
Female	9 (28%)	4 (57%)	5 (20%)	
Disease				0.15
TM	19 (59%)	3 (43%)	16 (64%)	
AML	6 (19%)	1 (14%)	5 (20%)	
ALL	3 (9.4%)	2 (29%)	1 (4.0%)	
WAS	2 (6.2%)	0 (0%)	2 (8.0%)	
MDS	1 (3.1%)	0 (0%)	1 (4.0%)	
HLH	1 (3.1%)	1 (14%)	0 (0%)	
HLA				0.10
Matched	14 (44%)	1 (14%)	13 (52%)	
Mismatched	18 (56%)	6 (86%)	12 (48%)	
Donor type				0.4
Relative	15 (47%)	2 (29%)	13 (52%)	
Unrelative	17 (53%)	5 (71%)	12 (48%)	
ATG used in conditioning				0.4
No	12 (38%)	4 (57%)	8 (32%)	
Yes	20 (62%)	3 (43%)	17 (68%)	
PTCY regimen				0.6
No	4 (12%)	0 (0%)	4 (16%)	
Yes	28 (88%)	7 (100%)	21 (84%)	
CB engraftment				0.2
No	22 (69%)	3 (43%)	19 (76%)	
Yes	10 (31%)	4 (57%)	6 (24%)	
Rituximab used in conditioning				>0.9
No	16 (50%)	4 (57%)	12 (48%)	
Yes	16 (50%)	3 (43%)	13 (52%)	
MNC [*10^8^/kg, median (IQR)]	20 (16, 28)	21 (20, 32)	20 (16, 27)	0.4
CD34^+^ [*10^6^/kg, median (IQR)]	10.6 (7.5, 12.9)	10.0 (6.7, 12.1)	10.6 (7.9, 13.4)	0.6
MNC of CB [*10^8^/kg, median (IQR)]	4.12 (3.18, 7.00)	4.43 (4.08, 6.28)	4.01 (2.54, 7.00)	0.7
CD34^+^ of CB (*10^5^/kg)	2.44 (1.49, 3.31)	1.90 (1.41, 3.07)	2.61 (1.90, 3.21)	0.2
Neutrophil recovery, days [median (IQR)]	21 (19, 26)	25 (19, 30)	21 (19, 24)	0.4
Platelet recovery, days [median (IQR)]	18 (13, 35)	39 (20, 56)	14 (13, 31)	0.14
Acute GVHD				0.023
None	18 (56%)	1 (14%)	17 (68%)	
Grade I–II	3 (9.4%)	1 (14%)	2 (8.0%)	
Grade III–IV	11 (34%)	5 (71%)	6 (24%)	
Chronic GVHD				>0.9
None or limited	26 (90%)	5 (100%)	21 (88%)	
Extensive	3 (10%)	0 (0%)	3 (12%)	
CMV infection				0.083
No	16 (50%)	1 (14%)	15 (60%)	
Yes	16 (50%)	6 (86%)	10 (40%)	
TA-TMA				>0.9
No	30 (94%)	7 (100%)	23 (92%)	
Yes	2 (6.2%)	0 (0%)	2 (8.0%)	
ITP				0.6
No	28 (88%)	7 (100%)	21 (84%)	
Yes	4 (12%)	0 (0%)	4 (16%)	
AIHA				0.7
No	13 (41%)	2 (29%)	11 (44%)	
Yes	19 (59%)	5 (71%)	14 (56%)	
Pneumonia				0.4
No	19 (59%)	3 (43%)	16 (64%)	
Yes	13 (41%)	4 (57%)	9 (36%)	
VOD				0.3
No	27 (84%)	5 (71%)	22 (88%)	
Yes	5 (16%)	2 (29%)	3 (12%)	
Hemorrhagic cystitis				0.073
No	20 (62%)	2 (29%)	18 (72%)	
Yes	12 (38%)	5 (71%)	7 (28%)	
Gastrointestinal hemorrhage				0.5
No	29 (91%)	6 (86%)	23 (92%)	
Yes	3 (9.4%)	1 (14%)	2 (8.0%)	
Indication of avatrombopag				0.029
PGF/SFPR	20 (62%)	7 (100%)	13 (52%)	
Promote engraftment	12 (38%)	0 (0%)	12 (48%)	
Megakaryocyte				0.004
Inadequate	5 (16%)	4 (57%)	1 (4.0%)	
Adequate	27 (84%)	3 (43%)	24 (96%)	
Survival status				0.042
Alive	30 (94%)	5 (71%)	25 (100%)	
Dead	2 (6.2%)	2 (29%)	0 (0%)	

AIHA, autoimmune hemolytic anemia; ALL, acute lymphoblast leukemia; AML, acute myeloid leukemia; ATG, antithymocyte globulin; CB, cord blood; CMV, cytomegalovirus; HLA, human leukocyte antigens; GVHD, graft-versus-host disease; HLH, hemophagocytic lymphohistiocytosis; ITP, immune thrombocytopenia; MDS, myelodysplastic syndromes; MNC, mononuclear cells; PGF, poor graft function; PTCY, post-transplantation cyclophosphamide; SFPR, secondary failure of platelet recovery; TA-TMA, transplant-associated thrombotic microangiopathy; TM, thalassemia major; VOD, veno-occlusive disease; WAS, wiskott–aldrich syndrome.

### Efficacy and safety of the treatment of avatrombopag

Based on the indication of avatrombopag, patients were divided into two groups (PGF/SFPR and engraftment-promotion) for further analysis (Table 2). The median day of initial avatrombopag treatment post-HSCT was at day +25. The median accumulative dosage of avatrombopag was 325 mg in PGF/SFPR group which was markedly higher than the engraftment-promotion group (*p* = 0.007). Nine (28%) patients received recombinant human thrombopoietin (rhTPO), and two patients were exposed to umbilical cord blood-derived mesenchymal stem cell (MSC).

**Table 2:** Features of avatrombopag treatment.

Characteristic	Overall, *N* = 32	PGF/SFPR, *N* = 20	Promote engraftment, *N* = 12	*p*-value
Initial day post-HSCT [days, median (IQR)]	25 (12, 49)	38 (25, 51)	10 (7, 12)	<0.001
Accumulative dosage [mg, median (IQR)]	205 (139, 430)	325 (188, 652)	160 (129, 202)	0.007
Duration [days, median (IQR)]	22 (16, 34)	31 (19, 43)	17 (15, 24)	0.006
Combination with rhTPO				0.4
No	23 (72%)	13 (65%)	10 (83%)	
Yes	9 (28%)	7 (35%)	2 (17%)	
Combination with MSC				0.5
No	30 (94%)	18 (90%)	12 (100%)	
Yes	2 (6.2%)	2 (10%)	0 (0%)	
Dose of platelet transfusion [U, median (IQR)]	2.0 (1.0, 4.2)	3.0 (1.0, 5.0)	1.2 (0.9, 2.5)	0.4
Dose of RBC transfusion [U, median (IQR)]	1.50 (0.75, 4.00)	1.50 (0.75, 3.62)	1.50 (0.75, 4.25)	>0.9
OR				0.3
No	3 (9.4%)	3 (15%)	0 (0%)	
Response	29 (91%)	17 (85%)	12 (100%)	
Time of OR [days, median (IQR)]	9 (4, 20)	16 (4, 44)	7 (3, 8)	0.003
CR				0.029
No	7 (22%)	7 (35%)	0 (0%)	
Yes	25 (78%)	13 (65%)	12 (100%)	
Time of CR [days, median (IQR)]	20 (10, 42)	30 (16, 61)	11 (9, 16)	0.002

CR, complete response; MSC, mesenchymal stem cell; OR, overall response; RBC, red blood cell; rhTPO, recombinant human thrombopoietin; U, unit.

**Figure 3:**
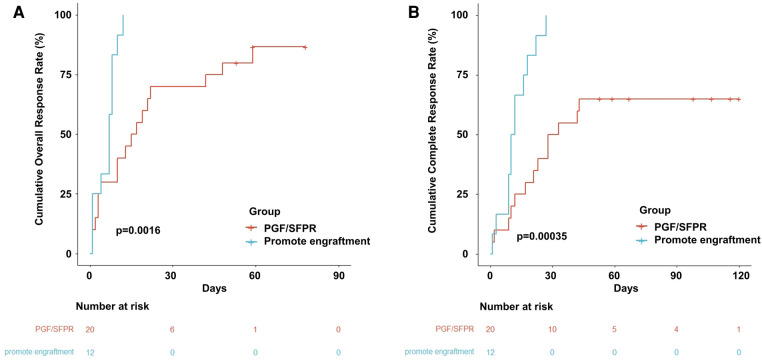
Cumulative event incidence. (**A**) Cumulative overall response rate. (**B**) Cumulative complete response rate.

The OR rate (ORR) and CR rate (CRR) in the entire population were 91% and 78%, respectively. Additionally, 17 (85%) patients and 13 (65%) patients achieved OR and CR, respectively in the PGF/SFPR group, while both ORR and CRR were 100% in the engraftment-promotion group (ORR *p* = 0.300 and CRR *p* = 0.029). Similarly, the cumulative ORR and CRR were significantly lower in PGF/SFPR group compared to the engraftment-promotion group (86.7% vs. 100%, *p* = 0.002 and 65.0% vs. 100%, *p* < 0.001, respectively) (Figure 3). The median time of OR achievement in the PGF/SFPR group was significantly longer than in the engraftment-promotion group 16 days vs. 7 days (*p* = 0.003). It required a predominantly longer time to achieve CR in PGF/SFPR group compared to the engraftment-promotion group (30 days vs. 11 days, *p* = 0.002). Surprisingly, the median dose of platelet and red blood cell (RBC) transfusion were 2 units and 1.5 units, respectively in the whole population.

**Figure 4:**
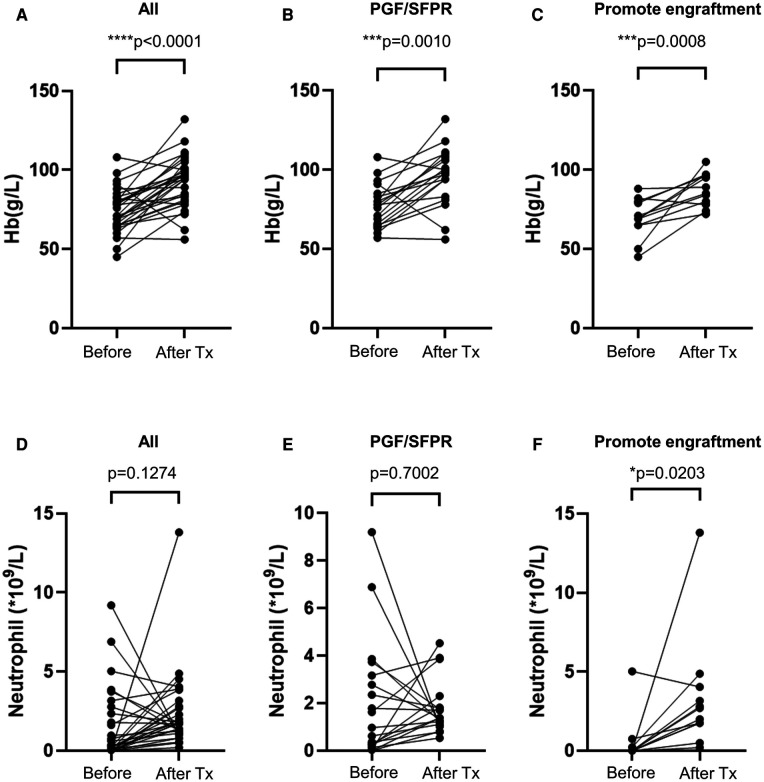
Avatrombopag affects hemoglobin and neutrophil level. (**A–C**) shows hemoglobin (Hb) level, while (**D–F**) shows the neutrophil level before treatment and after treatment (Tx). PGF, poor graft function; SFPR, secondary failure of platelet recovery. *p < 0.05, ***p < 0.001, ****p < 0.0001.

As result, the OS of the non-CR thrombocytopenia population dramatically dropped to 68.6% ± 18.6% [95% CI (4.03%–100%)] compared to the CR population which was 100% (*p* = 0.005) (Figure 2B). Interestingly, hemoglobin level was significantly elevated by avatrombopag in both PGF/SFPR and engraftment-promotion groups (*p* = 0.001 and *p* = 0.001, respectively), whereas neutrophil level was increased only in engraftment-promotion groups (*p* = 0.020) (Figure 4).

Grade II–III alanine aminotransferase increase occurred in three (9%) patients. All of these three cases were considered as not likely related to avatrombopag. No other adverse effect was found. Furthermore, no patients were intermitted or suspended from the avatrombopag treatment. In the end, the causes of the two dead patients were severe respiratory syncytial virus pneumonia and the progress of EB virus relative-hemophagocytic syndrome.

### Risk factors analyses for CR achievement of thrombocytopenia

Firstly, a statistically higher incidence of NR was found in both aGVHD (*p* = 0.023) and inadequate megakaryocyte status (*p* = 0.004) subgroups (Table 1). Secondly, in the univariate analysis (Table 3), grade III–IV aGVHD and inadequate megakaryocyte status were identified as risk factors (*p* = 0.03 and *p* = 0.01, respectively). Of note, late platelet recovery (*p* = 0.05), hemorrhagic cystitis (*p* = 0.05), and CMV infection (*p* = 0.06) were close to risk factors despite no statistical difference. Furthermore, both aGVHD and megakaryocyte status were further analyzed in multivariate analysis. Although inadequate megakaryocyte status potentially indicated non-CR achievement (*p* = 0.06), there was no significant difference in these two factors (Table 3).

**Table 3:** Univariate and multivariate analysis of risk factors affecting complete remission of avatrombopag.

Factors	Univariate analysis		Multivariate analysis	
	OR (95% CI)	*p*-value	OR (95% CI)	*p*-value
**Age**	0.89 (0.68–1.15)	0.37		
**Male vs. female**	0.19 (0.03–1.12)	0.07		
**Underlying disease**				
TM vs. AML	0.94 (0.08–11.15)	0.96		
TM vs. ALL	0.09 (0.01–1.39)	0.09		
TM vs. WAS	/	1		
TM vs. MDS	/	1		
TM vs. HLH	/	1		
**HLA matched vs. mismatched**	0.15 (0.02–1.47)	0.1		
**Relative donor vs. unrelative donor**	0.37 (0.06–2.27)	0.28		
**ATG not used vs. used**	2.83 (0.51–15.77)	0.23		
**PTCY not used vs. used**	/	1		
**CB not used vs. used**	0.24 (0.04–1.37)	0.11		
**Rituximab not used vs. used**	1.44 (0.27–7.83)	0.67		
**MNC, ×10^8^/kg**	0.96 (0.87–1.05)	0.35		
**CD34+, ×10^6^/kg**	1.05 (0.87–1.27)	0.59		
**MNC of CB, ×10^7^/kg**	0.92 (0.6–1.39)	0.68		
**CD34 + of CB, ×10^5^/kg**	1.37 (0.71–2.64)	0.34		
**Neutrophil recovery, day**	0.93 (0.82–1.06)	0.30		
**Platelet recovery, day**	0.96 (0.91–1)	0.05		
**Acute GVHD**				
None vs. I–II	0.12 (0.01–2.71)	0.18	0.12 (0.00–3.74)	0.18
None vs. III–IV	0.07 (0.01–0.73)	0.03	0.29 (0.01–8.33)	0.42
**Chronic GVHD**				
Non-extensive vs. extensive	/	0.99		
**Complications (no vs. yes)**				
CMV	0.11 (0.01–1.07)	0.06		
TA-TMA	/	1		
AIHA	0.51 (0.08–3.14)	0.47		
ITP	/	1		
Pneumonia	0.42 (0.08–2.32)	0.32		
VOD	0.34 (0.04–2.61)	0.3		
Hemorrhagic cystitis	0.16 (0.02–1)	0.05		
Gastrointestinal hemorrhage	0.52 (0.04–6.77)	0.62		
**PGF vs. SFPR**	2.67 (0.24–30.07)	0.43		
**Megakaryocyte status**				
Inadequate vs. adequate	32 (2.63–389.26)	0.01	20 (1.32, 862.18)	0.06
**RhTPO (no vs. yes)**	0.19 (0.03–1.12)	0.07		
**MSC (no vs. yes)**	/	1		

AIHA, autoimmune hemolytic anemia; ALL, acute lymphoblast leukemia; AML, acute myeloid leukemia; ATG, antithymocyte globulin; GVHD, graft-versus-host disease; CB, cord blood; CMV, cytomegalovirus; HLA, human leukocyte antigens; HLH, hemophagocytic lymphohistiocytosis; ITP, immune thrombocytopenia; MDS, myelodysplastic syndromes; MNC, mononuclear cells; PGF, poor graft function; PTCY, post-transplantation cyclophosphamide; SFPR, secondary failure of platelet recovery; TA-TMA, transplant-associated thrombotic microangiopathy; TM, thalassemia major; VOD, veno-occlusive disease; WAS, wiskott–aldrich syndrome.

## Discussion

To the best of our knowledge, this study was the first report to investigate the efficacy and safety of the avatrombopag for the treatment of post-HSCT thrombocytopenia in children's cohorts. Even though TPO-RAs, especially eltrombopag, were broadly evaluated in post-HSCT thrombocytopenia, only a few studies focused on pediatric patients (19, 20). Masetti et al. reported that the CRR was (8/9 patients) 88% using eltrombopag (20). A recent retrospective study, in which eltrombopag was used for PIT and SFPR pediatric patients, demonstrated that the ORR was (35/43 patients) 81.4% and the number of megakaryocytes in BM before eltrombopag treatment was identified as a predictor (19). Comparably, the ORR and CRR in PGF/SFPR setting were 85% and 65%, respectively in our study. In general, Mahat et al. summarized the ORR as 70% in adult PIT/SFPR setting from 13 studies (1). Moreover, the CRR was in a range of 50%–72% among PIT, SFPR, and PGF in adults (6). Regarding avatrombopag for post-HSCT thrombocytopenia, there were only a few studies published to date, and all of these studies were performed in only the adult population (18, 21). Zhou et al. reported that the ORR was 68.9% while CRR was 39.3% using avatrombopag for the treatment of delayed platelet engraftment and SFPR (18). Zhu et al. further investigated the effects of avatrombopag combined with MSC for post-HSCT thrombocytopenia. The result showed that the CRR was (13/16 patients) 81.3% (21). Therefore, the ORR and CRR of avatrombopag were comparable to eltrombopag in both adults and children.

The indication of the promotion of platelet engraftment was inspired by a prospective study in which the rhTPO was used to promote platelet engraftment (22). The study indicated that rhTPO promoted platelet engraftment and reduced the requirement for platelet transfusion. In addition, Wen et al. depicted that eltrombopag had a similar effect of engraftment-promotion to rhTPO (23). In our study, all patients in the engraftment-promotion group reached CR status, and only a median of 1.2 units platelet was required.

In previous studies, adequate megakaryocytes were consistently identified as a predictor of response to eltrombopag in both adults and children (19, 24). The potential mechanism may be related to the activation of various downstream signaling pathways, including JAK2/STAT5, PI3K/AKT, and ERK, eventually resulting in increased platelet production (25–27). Since the TPO receptor was widely expressed in megakaryocytes, adequate megakaryocytes provide sufficient responsible receptors for TPO-RA binding. Similarly, inadequate megakaryocytes were identified as a poor response factor in univariate analysis in our study. Furthermore, the TPO receptor was not only expressed in megakaryocytes but also in hematopoietic stem cells (28). Therefore, TPO-RA also played a critical role in severe aplastic anemia (26). This mechanism may explain why avatrombopag can elevate the levels of hemoglobin and partial neutrophil in our study.

Even though there were no markedly differences between avatrombopag alone and in the the combination with MSC or rhTPO, it still required a large cohort study to investigate further. On the one hand, the target of rhTPO and TPO-RA was not the same; hence it provided a hypothesis of synergistic effect from the combination of rhTPO and TPO-RA. On the other hand, severe GVHD may lead to prolong thrombocytopenia and no response to TPO-RA, which was shown in our univariate data. Since MSC can alleviate GVHD damage, avatrombopag was proven effective in combination with MSC in a recent study (21).

A meta-analysis study demonstrated that the pooled rate of adverse events was only 3% with no severe adverse events, in which increased alanine aminotransferase was the commonest adverse effect (29). Likely, besides three increased alanine aminotransferase events, no severe adverse event was reported in our study. It indicated that avatrombopag played a relatively safe role in this study.

Nevertheless, some limitations existed in our study including a relatively small cohort, common drawbacks of a retrospective study, and no negative control group. Additionally, the lack of durability data due to the short follow-up time, and the lack of control groups of the engraftment-promotion group in this study were the shortnesses. Thus, a large cohort of prospective controlled clinical trials were required in the future.

In conclusion, avatrombopag as an optional promising agent for post-HSCT thrombocytopenia promotes platelet recovery and engraftment in children patients.

## Data Availability

The original contributions presented in the study are included in the article/**Supplementary Material**, further inquiries can be directed to the corresponding author/s.
