# Supplementary material for: Avatrombopag for the treatment of thrombocytopenia in children's patients following allogeneic hematopoietic stem-cell transplantation: A pilot study
Source: Front Pediatr. 2023 Feb 15;11:1099372. doi: 10.3389/fped.2023.1099372 (PMC9975496; doi:10.3389/fped.2023.1099372)
Supplement: Supplementary file 1 [file Table1.docx]

Supplementary Table 1. Document of patients using avatrombopag.

| **Patient ID** | **Gender** | **Age (year)** | **Weight (kg)** | **Disease** | **HLA** | **Donor** | **STR engraftment** | **Complications post-transplant** | **Indication of avatrombopag** | **Combination** | **Maximum dosage** | **Adverse effect** | **Response** | **Outcome** |
| --- | --- | --- | --- | --- | --- | --- | --- | --- | --- | --- | --- | --- | --- | --- |
| 1 | male | 3 | 15 | AML | mismatched | unrelative | 100% donor (CB) | CMV infection, pneumonia | PGF | rhTPO | 10mg/d |  | CR | alive |
| 2 | male | 5 | 23 | ALL | mismatched | unrelative | 100% donor (CB) | aGVHD (III-IV), extensive cGVHD, TMA, AIHA, pneumonia, GI hemorrhage | PGF | MSC | 10mg/d |  | CR | alive |
| 3 | female | 10 | 33 | ALL | mismatched | unrelative | 100% donor (CB) | aGVHD (III-IV), pneumonia, VOD, HC | PGF | rhTPO | 20mg/d | ALT elevated (Grade II) | NR | dead |
| 4 | female | 6 | 19 | TM | mismatched | unrelative | 100% donor (CB) | aGVHD (III-IV), CMV infection, pneumonia, HC | PGF | rhTPO | 10mg/d |  | PR | alive |
| 5 | male | 11 | 36 | AML | mismatched | unrelative | 100% donor (CB) | aGVHD (III-IV), CMV infection, HC | PGF |  | 20mg/d | ALT elevated (Grade II) | CR | alive |
| 6 | female | 10 | 26.5 | AML | matched | relative | 100% donor | extensive cGVHD, CMV infection, TMA, AIHA | SFPR | rhTPO | 10mg/d |  | CR | alive |
| 7 | male | 5 | 15.2 | AML | mismatched | unrelative | 100% donor (CB) | aGVHD (III-IV), CMV infection, AIHA | PGF |  | 10mg/d |  | CR | alive |
| 8 | male | 3 | 12.7 | TM | matched | relative | 100% donor | CMV infection | PGF |  | 10mg/d |  | CR | alive |
| 9 | male | 2 | 9 | WAS | mismatched | unrelative | 100% donor (CB) | CMV infection, AIHA, pneumonia | promote engraftment | rhTPO | 10mg/d |  | CR | alive |
| 10 | male | 9 | 34 | HLH | matched | unrelative | 100% donor | aGVHD (III-IV), CMV infection, AIHA, HC | PGF | rhTPO | 20mg/d |  | PR | dead |
| 11 | male | 1 | 10 | AML | mismatched | unrelative | 100% donor (CB) | pneumonia | PGF |  | 10mg/d |  | CR | alive |
| 12 | male | 2 | 12 | TM | matched | unrelative | 100% donor | pneumonia | SFPR |  | 10mg/d |  | CR | alive |
| 13 | male | 8 | 19.2 | TM | mismatched | relative | 100% donor | aGVHD (I-II), extensive cGVHD, CMV infection, AIHA, VOD, HC | PGF | MSC | 10mg/d |  | CR | alive |
| 14 | male | 5 | 15 | ALL | mismatched | unrelative | 100% donor (CB) | aGVHD (III-IV), CMV infection, AIHA, pneumonia, HC | PGF | rhTPO | 15mg/d |  | NR | alive |
| 15 | male | 4 | 14 | TM | matched | unrelative | 100% donor | AIHA, pneumonia | SFPR | rhTPO | 10mg/d |  | CR | alive |
| 15 | male | 4 | 14 | TM | matched | unrelative | 100% donor | AIHA | SFPR |  | 10mg/d |  | CR | alive |
| 16 | female | 7 | 16.3 | TM | mismatched | relative | 100% donor | CMV infection, AIHA, VOD | SFPR |  | 10mg/d | ALT elevated (Grade III) | PR | alive |
| 17 | female | 9 | 20 | TM | matched | unrelative | 100% donor | AIHA, pneumonia | promote  engraftment | | 10mg/d |  | CR | alive |
| 18 | male | 5 | 14 | WAS | mismatched | relative | 100% donor | CMV infection, AIHA | PGF |  | 10mg/d |  | CR | alive |
| 19 | male | 11 | 19 | TM | matched | unrelative | 100% donor | ITP, AIHA | promote  engraftment | | 10mg/d |  | CR | alive |
| 20 | female | 8 | 25 | TM | matched | relative | 100% donor | ITP, AIHA | promote  engraftment | | 10mg/d |  | CR | alive |
| 21 | male | 13 | 32 | TM | matched | unrelative | 100% donor | aGVHD (III-IV), ITP, AIHA, VOD, HC | promote engraftment | rhTPO | 20mg/d |  | CR | alive |
| 22 | male | 3 | 18 | TM | mismatched | relative | 100% donor | aGVHD (III-IV), CMV infection, AIHA, pneumonia | PGF |  | 10mg/d |  | CR | alive |
| 22 | male | 3 | 17.8 | TM | mismatched | relative | 100% donor | aGVHD (III-IV), HC, GI hemorrhage | promote engraftment | | 10mg/d |  | CR | alive |
| 23 | male | 9 | 19.1 | TM | matched | relative | 100% donor | ITP, AIHA, VOD, HC | promote engraftment | | 10mg/d |  | CR | alive |
| 24 | male | 4 | 14.5 | TM | mismatched | relative | 100% donor | aGVHD (I-II), CMV infection, AIHA, pneumonia | PGF |  | 20mg/d |  | PR | alive |
| 25 | female | 4 | 12.5 | TM | matched | relative | 100% donor | pneumonia | promote engraftment | | 10mg/d |  | CR | alive |
| 26 | male | 10 | 22 | TM | matched | relative | 100% donor | HC | promote engraftment | | 10mg/d |  | CR | alive |
| 27 | female | 10 | 20.2 | TM | matched | relative | 100% donor | CMV infection, HC | promote engraftment | | 10mg/d |  | CR | alive |
| 28 | male | 4 | 16.5 | TM | mismatched | relative | 100% donor | aGVHD (I-II) | promote engraftment | | 10mg/d |  | CR | alive |
| 29 | female | 11 | 36.4 | AML | mismatched | unrelative | 100% donor (CB) | aGVHD (III-IV), CMV infection, AIHA, HC, GI hemorrhage | PGF |  | 40mg/d |  | NR | alive |
| 30 | male | 7 | 19 | MDS | mismatched | relative | 100% donor | ITP before transplant | promote engraftment | | 10mg/d |  | CR | alive |

AIHA, autoimmune hemolytic anemia; ALL, acute lymphoblast leukemia; AML, acute myeloid leukemia; CB, cord blood; CMV, cytomegalovirus; CR, complete remission; GVHD, graft-versus-host disease; HC, hemorrhagic cystitis; HLA, human leukocyte antigens; HLH, Hemophagocytic lymphohistiocytosis; ITP, Immune thrombocytopenia; MDS, Myelodysplastic syndromes; NR, no response; PGF, Poor graft function; PR, partial response; PTCY, post-transplantation cyclophosphamide; SFPR, secondary failure of platelet recovery; TMA, thrombotic microangiopathy; TM, thalassemia major; VOD, veno-occlusive disease; WAS, Wiskott-Aldrich syndrome.
